# Dehydroquinate dehydratase/shikimate dehydrogenases involved in gallate biosynthesis of the aluminum-tolerant tree species *Eucalyptus camaldulensis*

**DOI:** 10.1007/s00425-020-03516-w

**Published:** 2020-12-21

**Authors:** Ko Tahara, Mitsuru Nishiguchi, Evelyn Funke, Shin-Ichi Miyazawa, Takafumi Miyama, Carsten Milkowski

**Affiliations:** 1 Interdisciplinary Center for Crop Plant Research, Martin-Luther University Halle-Wittenberg, Hoher Weg 8, 06120 Halle, Germany; 2 Department of Forest Molecular Genetics and Biotechnology, Forestry and Forest Products Research Institute, 1 Matsunosato, Tsukuba, Ibaraki 305-8687 Japan; 3 Department of Bioorganic Chemistry, Leibniz Institute of Plant Biochemistry, Weinberg 3, 06120 Halle, Germany; 4 Department of Disaster Prevention, Meteorology and Hydrology, Forestry and Forest Products Research Institute, 1 Matsunosato, Tsukuba, Ibaraki 305-8687 Japan; 5 Present Address: AGRIPOLY: International Graduate School in Agricultural and Polymer Sciences, Martin Luther University Halle-Wittenberg, Betty-Heimann-Straße 3, 06120 Halle, Germany

**Keywords:** Aluminum resistance, Biosynthetic pathway, Gallic acid, Hydrolyzable tannin, Quinate dehydrogenase, Shikimate pathway

## Abstract

**Main conclusion:**

*Eucalyptus camaldulensis* EcDQD/SDH2 and 3 combine gallate formation, dehydroquinate dehydratase, and shikimate dehydrogenase activities. They are candidates for providing the essential gallate for the biosynthesis of the aluminum-detoxifying metabolite oenothein B.

**Abstract:**

The tree species *Eucalyptus camaldulensis* shows exceptionally high tolerance against aluminum, a widespread toxic metal in acidic soils. In the roots of *E. camaldulensis*, aluminum is detoxified via the complexation with oenothein B, a hydrolyzable tannin. In our approach to elucidate the biosynthesis of oenothein B, we here report on the identification of *E. camaldulensis* enzymes that catalyze the formation of gallate, which is the phenolic constituent of hydrolyzable tannins. By systematical screening of *E. camaldulensis* dehydroquinate dehydratase/shikimate dehydrogenases (EcDQD/SDHs), we found two enzymes, EcDQD/SDH2 and 3, catalyzing the NADP^+^-dependent oxidation of 3-dehydroshikimate to produce gallate. Based on extensive in vitro assays using recombinant EcDQD/SDH2 and 3 enzymes, we present for the first time a detailed characterization of the enzymatic gallate formation activity, including the cofactor preferences, pH optima, and kinetic constants. Sequence analyses and structure modeling suggest the gallate formation activity of EcDQD/SDHs is based on the reorientation of 3-dehydroshikimate in the catalytic center, which facilitates the proton abstraction from the C5 position. Additionally, EcDQD/SDH2 and 3 maintain DQD and SDH activities, resulting in a 3-dehydroshikimate supply for gallate formation. In *E. camaldulensis*, *EcDQD/SDH2* and *3* are co-expressed with *UGT84A25a/b* and *UGT84A26a/b* involved in hydrolyzable tannin biosynthesis. We further identified EcDQD/SDH1 as a “classical” bifunctional plant shikimate pathway enzyme and EcDQD/SDH4a/b as functional quinate dehydrogenases of the NAD^+^/NADH-dependent clade. Our data indicate that in *E. camaldulensis* the enzymes EcDQD/SDH2 and 3 provide the essential gallate for the biosynthesis of the aluminum-detoxifying metabolite oenothein B.

**Supplementary Information:**

The online version contains supplementary material available at 10.1007/s00425-020-03516-w.

## Introduction

In plants and microorganisms, the shikimate pathway produces chorismate, the metabolic precursor of aromatic amino acids, and is therefore essential for protein biosynthesis. Moreover, all shikimate pathway intermediates can contribute to the synthesis of specialized compounds (Herrmann and Weaver 1999; Maeda and Dudareva 2012). Recent studies in plants suggest that shikimate dehydrogenases (SDHs, EC 1.1.1.25) might help to link the shikimate pathway to the formation of ecologically important natural products (Guo et al. 2014; Bontpart et al. 2016).

**Fig. 1:**
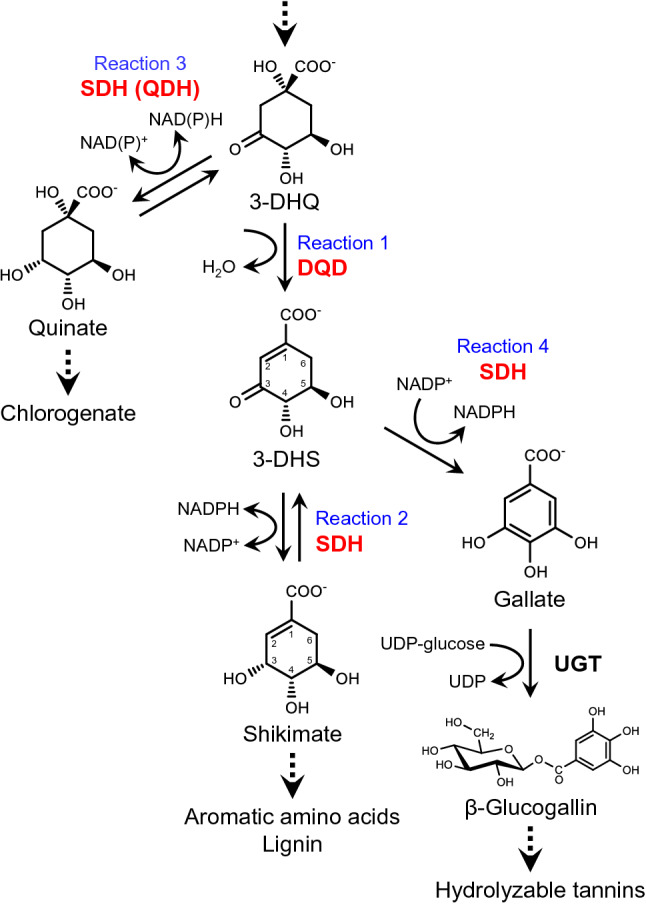
Shikimate, quinate, and gallate biosynthesis catalyzed by DQD/SDH family enzymes. Plant DQD/SDHs are proposed to link the shikimate pathway to gallate and quinate metabolism. 3-DHQ, 3-dehydroquinate; 3-DHS, 3-dehydroshikimate; DQD, dehydroquinate dehydratase; QDH, quinate dehydrogenase; SDH, shikimate dehydrogenase; UGT, UDP glycosyltransferase

Plant SDH enzymes are fused to dehydroquinate dehydratases (DQDs, EC 4.2.1.10) to form bifunctional DQD/SDH enzymes (Bischoff et al. 2001; Peek and Christendat 2015). In the shikimate pathway, “classical” DQD/SDH enzymes catalyze two successive reactions (Fig. 1), the dehydration of 3-dehydroquinate (3-DHQ) to 3-dehydroshikimate (3-DHS) (reaction 1, DQD activity) and the reversible reduction of 3-DHS to shikimate (reaction 2, SDH activity). Genome analyses revealed that many seed plants contain genes for multiple DQD/SDH enzymes (Carrington et al. 2018 ; Huang et al. 2019). In some of these plant species, diverse DQD/SDH functions were observed. In *Populus trichocarpa* (Pot), two DQD/SDH enzymes with quinate dehydrogenase (QDH) activity have been identified (PotQDH1 and PotQDH2) (Guo et al. 2014). These enzymes catalyze the reversible reduction of 3-DHQ to quinate (reaction 3, QDH activity) (Fig. 1) and exhibit only residual SDH activity. *In planta*, they may link the shikimate pathway to the synthesis of chlorogenate, the caffeoyl ester of quinate, which accumulates in several species (Bentley 1990). Phylogenetic analyses revealed that PotQDH1 and PotQDH2 define a distinct phylogenetic cluster within the DQD/SDH family (Carrington et al. 2018).

*Juglans regia* and *Vitis vinifera* contain DQD/SDH enzymes that catalyze the oxidation of 3-DHS to gallate (reaction 4, gallate formation activity) (Fig. 1) (Muir et al. 2011; Bontpart et al. 2016). In many dicotyledonous plant species, gallate is conjugated via its energy-rich 1-*O*-glucose ester β-glucogallin to various acceptor molecules leading to the accumulation of polyphenols like hydrolyzable tannins (gallotannins and ellagitannins), condensed tannins (galloylated proanthocyanidins) or galloylated catechins (Fig. 1) (Haslam and Cai 1994; Niemetz and Gross 2005; Liu et al. 2012).

Because of their protein-complexing and astringent properties, hydrolyzable tannins were traditionally associated with the chemical defense of plants against herbivores (Barbehenn and Constabel 2011). Our recent work on the extremely aluminum (Al)-tolerant tree species *Eucalyptus camaldulensis* added a novel ecologically important function to the known effects of hydrolyzable tannins (Tahara et al. 2014, 2017; Zhang et al. 2016). We determined that *E. camaldulensis* roots accumulate large amounts of the hydrolyzable tannin oenothein B and that the binding of Al^3+^ by oenothein B in the root symplast contributes to the considerable Al tolerance of this tree species. Aluminum toxicity is a major abiotic stress factor that limits the productivity of plants growing in acidic soils, which comprise approximately 30% of the ice-free land area worldwide (von Uexküll and Mutert 1995; Kochian et al. 2015). Therefore, strategies to increase the Al tolerance of crops and trees are required for sustainable food and forest production.

As part of our long-term approach to engineer Al tolerance in crops and trees, we started with the identification and molecular characterization of key hydrolyzable tannin biosynthetic enzymes in *E. camaldulensis.* We previously identified the UDP glucosyltransferases UGT84A25a/b and UGT84A26a/b, which catalyze the synthesis of β-glucogallin (Fig. 1) (Tahara et al. 2018).

We herein describe the isolation and systematic characterization of DQD/SDH family proteins from *E. camaldulensis* to identify gallate-forming enzymes that may link the shikimate pathway to hydrolyzable tannin biosynthesis. Following homology-based cDNA cloning, heterologous protein production in *Escherichia coli*, and in vitro assays of the catalytic activities of the highly purified enzymes, we identified four *E. camaldulensis* DQD/SDH family proteins (EcDQD/SDH1, 2, 3, and 4a). Specifically, EcDQD/SDH2 and 3 primarily catalyzed the formation of gallate (reaction 4) (Fig. 1), whereas EcDQD/SDH1 was characterized as a “classical” DQD/SDH enzyme (reactions 1 and 2) and EcDQD/SDH4a (EcQDHa) exhibited QDH activity (reaction 3). The gallate-forming enzymes EcDQD/SDH2 and 3 were extensively analyzed in in vitro assays. We present herein for the first time the kinetic constants as well as data regarding substrate and cofactor preferences, the effects of pH, and the influence of divalent metal ions for the following three catalytic activities of gallate-forming DQD/SDH enzymes: gallate formation activity (reaction 4), DQD activity (reaction 1), and SDH activity (reaction 2). To predict the physiological role of the distinct DQD/SDH enzymes *in planta*, we analyzed the organ-specific and Al-dependent expression of EcDQD/SDH genes and checked for co-expression. Furthermore, sequence analyses, phylogenetic classification, and structure modeling revealed the molecular basis of the substrate and reaction specificities in the DQD/SDH enzyme family.

## Materials and methods

### Plant materials

A *Eucalyptus camaldulensis* Dehnh. clone (Myrtaceae; seed lot 19,708; Australian Tree Seed Centre, CSIRO) was propagated by cutting, and cultured hydroponically in a growth chamber as previously described (Tahara et al. 2018). The youngest fully expanded leaves, stems, and roots were sampled from uniformly growing plantlets (approximately 20 cm tall) and frozen in liquid nitrogen.

Plantlets were treated with Al as previously described (Tahara et al. 2014). The long-term treatment involved exposing the roots to a nutrient solution containing 0 or 1.5 mM AlCl_3_ (pH 4) for 5 days. The short-term treatment was performed by exposing roots to 0.35 mM CaCl_2_ solution containing 0 or 1 mM AlCl_3_ (pH 4) for 24 h. After the Al treatments, the 2-cm apical portion of the root was excised and frozen in liquid nitrogen.

### RNA extraction

Total RNA was extracted from *E. camaldulensis* using the hexadecyltrimethylammonium bromide method and purified as previously described (Tahara et al. 2018).

### cDNA cloning

The *EcDQD/SDH* cDNAs were cloned as previously described (Tahara et al. 2018). Briefly, the first-strand cDNA was synthesized from root total RNA and used to amplify target cDNAs by PCR using specific primer sets (Table S1). The cDNAs were ligated into the pBluescript II SK( +) vector and sequenced. The DNA sequences were analyzed with GENETYX version 13 (GENETYX) and submitted to the DNA Data Bank of Japan. Protein sequences were analyzed with InterPro (http://www.ebi.ac.uk/interpro/) (Mitchell et al. 2018) to predict functional domains. The subcellular localization of proteins was predicted with WoLF PSORT (https://wolfpsort.hgc.jp/) (Horton et al. 2007). The phylogenetic analysis of DQD/SDH sequences was conducted with MEGA7 (Kumar et al. 2016).

### Heterologous expression and purification of EcDQD/SDHs

Recombinant EcDQD/SDHs were produced as N-terminal glutathione *S*-transferase (GST)-tagged proteins and affinity-purified (Khater et al. 2012). The *EcDQD/SDH* coding sequences lacking their start codons were amplified from the cloned cDNAs with the Phusion High-Fidelity PCR Master Mix (New England BioLabs) and specific primers (Table S1) designed for the restriction cloning of the PCR products into the expression vector pGEX-4 T-2 (GE Healthcare). Recombinant plasmids were verified by DNA sequencing and used to transform *E. coli* BL21-CodonPlus(DE3)-RIL cells (Agilent Technologies). To produce proteins, cells were grown at 37 °C in 2 × YT medium supplemented with 100 µg ml^−1^ ampicillin and 50 µg ml^−1^ chloramphenicol until the early logarithmic phase (OD_600_ 0.8) and then induced by isopropyl β-d-thiogalactopyranoside (0.1 mM) for 20 h at 20 °C. Cells were harvested by centrifugation and stored at − 80 °C.

For batch purification of proteins, cells were lysed by sonication in phosphate-buffered saline (pH 7.4), 0.1% (v/v) β-mercaptoethanol, 1% (v/v) Triton X-100, and 1 mg ml^−1^ lysozyme. After centrifugation, the supernatant containing the GST-tagged proteins was incubated with Glutathione Sepharose 4B (GE Healthcare) for 1 h at room temperature. Unspecific proteins were removed by washing the resin three times with phosphate-buffered saline containing 0.1% (v/v) β-mercaptoethanol. Recombinant EcDQD/SDH proteins were cleaved from their GST tags with thrombin. The purified proteins were finally dissolved in phosphate-buffered saline containing 16% (v/v) glycerol and stored at − 20 °C. Protein concentrations were determined with the Qubit Protein Assay Kit (Thermo Fisher Scientific). Protein integrity was confirmed by 12% SDS-PAGE (e-PAGEL HR, ATTO; Fig. S1).

### Identification of enzymatic reaction products by HPLC

Recombinant EcDQD/SDHs (1.25–375 µg ml^−1^) were incubated with 5 mM substrate (3-DHQ, 3-DHS, shikimate, or quinate) and 5 mM cofactor (NADPH, NADP^+^, NADH, NAD^+^, or none) in 100 mM Bis–tris propane HCl buffer (pH 6.5–9.5; at the optimal pH for each reaction) at 30 °C for 10 min. The reaction was stopped by adding 0.25 volumes of 11.5 M HCl.

Reaction products were analyzed with the 1100 Series HPLC system (Agilent Technologies). The HPLC conditions (Guo et al. 2014) were as follows: column, Aminex HPX-87H Column (300 × 7.8 mm, particle size 9 µm; Bio-Rad); column temperature, 35 °C; eluent, 10 mM H_2_SO_4_ in water; flow rate, 0.6 ml min^−1^; and detection, absorbance at 215 nm (shikimate, quinate, and 3-DHQ), 235 nm (3-DHS), and 270 nm (gallate). The reaction products were identified based on a comparison with authentic standards regarding their retention times and UV absorption spectra.

### Validation of enzymatic reaction products by GC–MS

Enzymatic reaction products were further validated by GC–MS analysis based on a previously described method (Fiehn et al. 2000). Aliquots of the reaction mixtures and standard solution were desiccated by freeze-drying. Ketone groups were methoxylated in 10 µl 40 mg ml^−1^ methoxyamine hydrochloride in pyridine at 30 °C for 90 min. Acidic protons were then trimethylsilylated in 90 µl MSTFA plus 1% TMCS (Thermo Fisher Scientific) for 30 min at 37 °C. Samples were analyzed with a GC–MS system (7890B/5977C, Agilent Technologies), with the following conditions: injection volume, 1 µl; split ratio, 10:1; injection temperature, 250 °C; column, DB-5MS (30 m × 0.25 mm i.d., 0.25 µm film thickness; Agilent J&W); column temperature, 60 °C (0–1 min), 60–325 °C (1–27.5 min, increased at 10 °C min^−1^), and 325 °C (27.5–37.5 min); carrier gas, helium; constant flow rate, 0.841 ml min^−1^; interface temperature, 290 °C; ion source temperature, 250 °C; and quadrupole temperature, 150 °C. Reaction products were identified based on a comparison with authentic standards regarding their retention times and mass spectra.

### Enzyme activities of four EcDQD/SDHs

To assess the enzymatic activities of the four EcDQD/SDHs, each recombinant enzyme (0.0625–125 µg ml^−1^) was assayed under standard conditions (Table S2) with different substrate and cofactor combinations (both at saturating concentrations) at the optimal pH and 30 °C. The pH was controlled with 100 mM Bis–tris propane HCl buffer (pH 6.5–9) or 200 mM glycine–NaOH buffer (pH 10.5). For the gallate-forming assay, 8 mM ascorbic acid was added to the reaction mixture to prevent gallate degradation (Bontpart et al. 2016). For shikimate and quinate formation or oxidation assays, a decrease or increase in NAD(P)H levels (extinction coefficient, 6.22 mM^−1^ cm^−1^) was measured spectrophotometrically by monitoring the absorbance at 340 nm every 10 s for 120 s with a microplate reader (SpectraMax 340PCS, Molecular Devices). For DQD and gallate- and quinate-forming assays, the reaction was stopped by mixing aliquots of the mixture with 0.25 volumes of 11.5 M HCl after incubations of 40, 80, and 120 s (DQD and quinate-forming assays) or 200, 400, and 600 s (gallate-forming assay). The 3-DHS, gallate, and quinate contents were then measured by HPLC as described below. Enzyme activities were calculated based on the linear increase in the reaction products or the linear increase or decrease in NAD(P)H levels over reaction times of 120 s or 600 s. In the gallate-forming assay, a reaction mixture lacking enzymes was also incubated and analyzed for gallate production because of the observed spontaneous formation of gallate at high pH (Ossipov et al. 2003; Bontpart et al. 2016). The enzymatic production of gallate was determined by subtracting the spontaneously produced gallate (i.e., without enzymes) from the total gallate produced with enzymes.

Gallate formation activity was determined based on gallate production and not NADPH production because the NADPH formed by the gallate formation activity can be consumed by shikimate formation activity. Similarly, the quinate formation activity of EcDQD/SDH1–3 in the presence of NADPH was determined based on quinate production and not NADPH consumption because the reactions from 3-DHQ to shikimate through 3-DHS also consume NADPH.

### pH and metal ion effects on enzyme activities and kinetic parameters

Each enzyme was assayed under standard conditions (Table S2) unless otherwise noted. The effect of pH was examined at pH 7.5–11 for the gallate-forming assay and pH 6.5–9.5 for the other assays. The buffers for the assays were 100 mM Bis–tris propane HCl buffer (pH 7.5–9.5) or 200 mM glycine–NaOH buffer (pH 10–11). We used NADPH/NADP^+^ as a cofactor for the shikimate formation and oxidation assays and the gallate-forming assay, whereas NADH/NAD^+^ was used for the quinate formation and oxidation assays.

To test the effect of divalent metal ions on the gallate formation activity, recombinant EcDQD/SDH2 or 3 was assayed with 5 mM divalent metal (chloride salt) or EDTA-Na_2_ in 10 mM 3-DHS, 6 mM NADP^+^, 8 mM ascorbic acid, and 200 mM glycine–NaOH buffer (pH 10).

Kinetic parameters for each substrate or cofactor were determined with at least eight concentrations at the optimal pH. The *V*_max_ and *K*_m_ values were obtained via the nonlinear fitting of the assay data to the Michaelis–Menten equation based on the Levenberg–Marquardt algorithm of KaleidaGraph version 4.5 (Hulinks).

### Quantification of enzymatic reaction products by HPLC

The HPLC conditions with the Aminex HPX-87H Column described above were used to quantify the 3-DHS formed by the DQD activity. To quantify the gallate content, the following HPLC conditions were applied: column, Shim-pack CLC-ODS(M) (250 × 4.6 mm, particle size 5 µm; Shimadzu); column temperature, 30 °C; eluent A, 0.1% (v/v) formic acid in water; eluent B, 0.1% (v/v) formic acid in acetonitrile; flow rate, 1 ml min^−1^; and detection, absorbance at 270 nm. The column was eluted isocratically with 3% eluent B. The 3-DHS and gallate concentrations were determined by comparing the peak areas of the reaction mixture and the standard.

### Quantitative real-time RT-PCR

First-strand cDNA was synthesized from 1 µg total RNA, and a quantitative real-time PCR assay with specific primer sets (Table S1) was completed with the SsoAdvanced SYBR Green Supermix (Bio-Rad) as previously described (Tahara et al. 2018). Gene expression levels were normalized against the *EcActin* expression level (Sawaki et al. 2013).

### Metabolite content in *Eucalyptus camaldulensis*

Metabolites were extracted as previously described (Okazaki et al. 2010), with some modifications. The sample (50 mg) was ground and then mixed with 1 ml ice-cold methanol:chloroform:water (5:2:2, by vol.) and 20 µl internal standard solution (0.2 mg ml^−1^ ribitol in water) for 90 min at 4 °C in darkness. After centrifugation, 900 µl methanol/water supernatant was added to 400 µl water, vigorously mixed, and centrifuged. The supernatant was concentrated in a centrifugal evaporator to remove methanol and subsequently desiccated by freeze-drying. The dried residue was analyzed by GC–MS as described above. Each metabolite in the sample was quantified by comparing the peak area with that of an authentic standard.

### Structure modeling of EcDQD/SDH1–3

Homology models of EcDQD/SDH1–3 were generated with YASARA version 18.3.19 (Krieger et al. 2009; Krieger and Vriend 2014, 2015) using the hm_build.mcr macro (www.yasara.org/macros.htm). For these enzymes, homology models were built based on different crystal structures of AtDQD/SDH (Uniprot entry: Q9SQT8). Because of the transfer of the co-crystallized product shikimate and the co-factor NADP^+^ during the modeling, templates based on the PDB entry 2O7S (Singh and Christendat 2007) were chosen for further analysis. After selecting the model, the substrate 3-DHS was docked into the active site of the models as well as the crystal structure of PDB entry 2O7S using the dock tool of MOE 2019.0101 (Molecular Operating Environment, Chemical Computing Group ULC 2019). Specifically, NADP^+^ and all amino acids within 4.5 Å of the shikimate molecule were selected as the docking site. One hundred poses were generated with the Triangle Matcher placement method and ranked according to the Affinity dG score. Models were refined based on Induced Fit scored by GBVI/WSA dG to create 30 final poses that were analyzed according to their orientation in the binding pocket. For each enzyme, the selected final substrate poses substantially overlapped with the original position of the co-crystallized shikimate, with an orientation leading to the production of gallate or shikimate.

As a final refinement step, a molecular dynamics simulation (MDS) was run in YASARA using the md_refine.mcr macro (www.yasara.org/macros.htm). During the simulation, the energy minimized protein–ligand complex was set in an explicit water box at physiological pH (7.4) and NaCl concentration (0,9%) and the simulation was run at 298 K. The YASARA2 force field (Krieger et al. 2009) was applied and during the simulation, the force field parameters were iteratively adjusted until the damage done to the structures was minimal. The duration of the MDS was 1 ns and a snapshot of the current model was saved every 25 ps and subsequently, energy minimized. During all refinement stages, the generated models were analyzed regarding their folding behavior and protein geometry with ProSA (Sippl 1993; Wiederstein and Sippl 2007) and PROCHECK (Laskowski et al. 1993). The MDS snapshot with the best protein geometry was selected for the comparison of the active sites after the final refinement. The final models were of high quality, with over 94% of the residues in the most favorable region of the Ramachandran plot (AtDQD/SDH: 94.3%, EcDQD/SDH1: 94.9%, EcDQD/SDH2: 95.7%, and EcDQD/SDH3: 96.0%). Only two residues resided in disallowed regions for EcDQD/SDH1 (Lys25 and Ala383), both of which were located in loop regions on the protein surface and far from the substrate-binding sites. The overall knowledge-based energy of all models calculated for a window size of 40 was less than 0.

### Statistical analysis

Enzyme kinetic parameters were obtained by repeating the assay four times and expressed as the estimate ± SE. The values of enzyme activities, gene expression, and metabolite concentrations were presented as the mean ± SD from at least three replicates. The data were analyzed using Student’s *t* test, Dunnett’s test, the Tukey–Kramer test, or Pearson’s correlation test with BellCurve for Excel version 3.21 (Social Survey Research Information).

## Results

### Cloning of *E. camaldulensis* DQD/SDH genes

On the basis of homology with *Arabidopsis thaliana* (At) DQD/SDH sequences, we selected four candidate DQD/SDH genes (EcC055014.20, EcC035206.10, EcC054875.120, and EcC015288.50) from the *E. camaldulensis* genome database (http://www.kazusa.or.jp/eucaly/). Because EcC015288.50 was represented by two closely related sequence variants, our homology-based RT-PCR cloning approach resulted in the isolation of five full-length candidate cDNAs from *E. camaldulensis* roots. Sequence-based functional predictions with the InterPro database indicated that all five encoded candidate proteins contain DQD and SDH domains (Fig. S2). Hence, they were designated as EcDQD/SDH1 (Accession No. LC487988), EcDQD/SDH2 (LC487989), EcDQD/SDH3 (LC487990), EcDQD/SDH4a (LC487991), and EcDQD/SDH4b (LC487992).

**Fig. 2:**
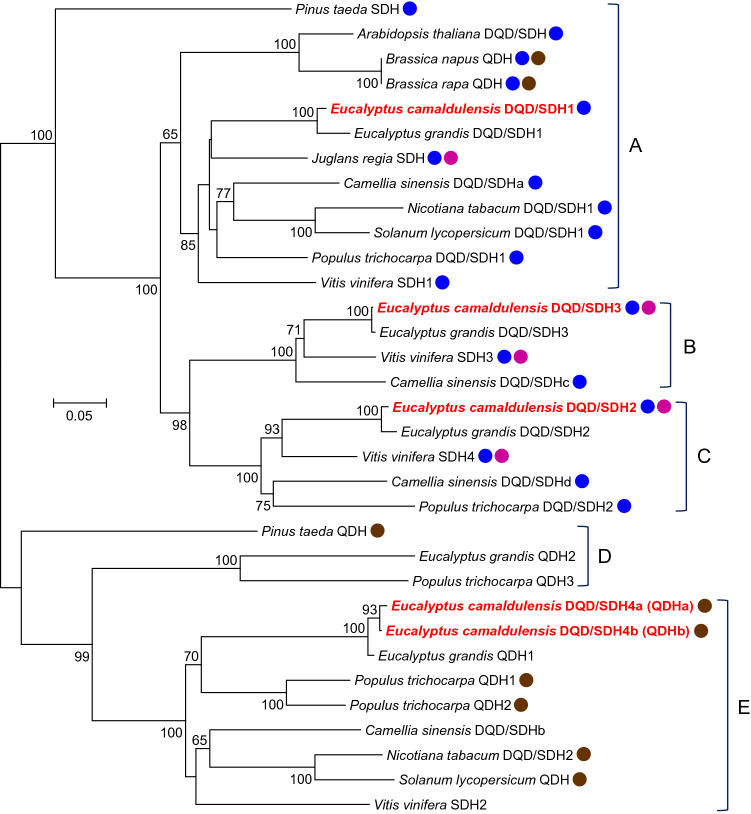
Phylogenetic analysis of five EcDQD/SDHs and other functionally characterized DQD/SDH family members in seed plants. The phylogenetic tree was constructed based on an alignment of multiple full-length protein sequences according to the neighbor-joining method. The scale bar represents 0.05 fixed mutations per site. Bootstrap values (1000 replicates) greater than 60% are indicated. The accession numbers of the DQD/SDHs and QDHs are listed in Table S3. The EcDQD/SDHs are highlighted in red letters. Magenta dots indicate enzymes with gallate formation activity. Blue and brown dots indicate enzymes mainly exhibiting SDH and QDH activities, respectively

The phylogenetic analysis of the five EcDQD/SDHs enabled us to predict catalytic activities. The DQD/SDH enzymes of seed plants form five phylogenetic groups assigned to two major clades (Bontpart et al. 2016). The clade containing groups A–C consists of enzymes with SDH and/or gallate formation activities, whereas the clade built from groups D and E comprises enzymes exhibiting QDH activity (Fig. 2; Table S3). As an exception, two *Brassica* enzymes combined SDH and QDH activities. Thus, we predicted that EcDQD/SDH1, 2, and 3 exhibit SDH and/or gallate formation activities, whereas EcDQD/SDH4a and 4b have QDH activity. Because the *EcDQD/SDH4a* and *EcDQD/SDH4b* genes may represent allelic variants encoding enzymes with 99.2% amino acid identity, only EcDQD/SDH4a was included in subsequent analyses.

### Enzymatic reaction specificity of four EcDQD/SDHs

To characterize their enzymatic functions, EcDQD/SDH1–4a were expressed in *E. coli* as recombinant N-terminal GST-tagged proteins and affinity-purified (Fig. S1). After removing the GST tag, the native proteins were used for in vitro enzyme assays. The identity of all enzymatic reaction products was confirmed by comparisons with authentic standards using a combination of HPLC (Fig. S3) and GC–MS (Fig. S4).

**Fig. 3:**
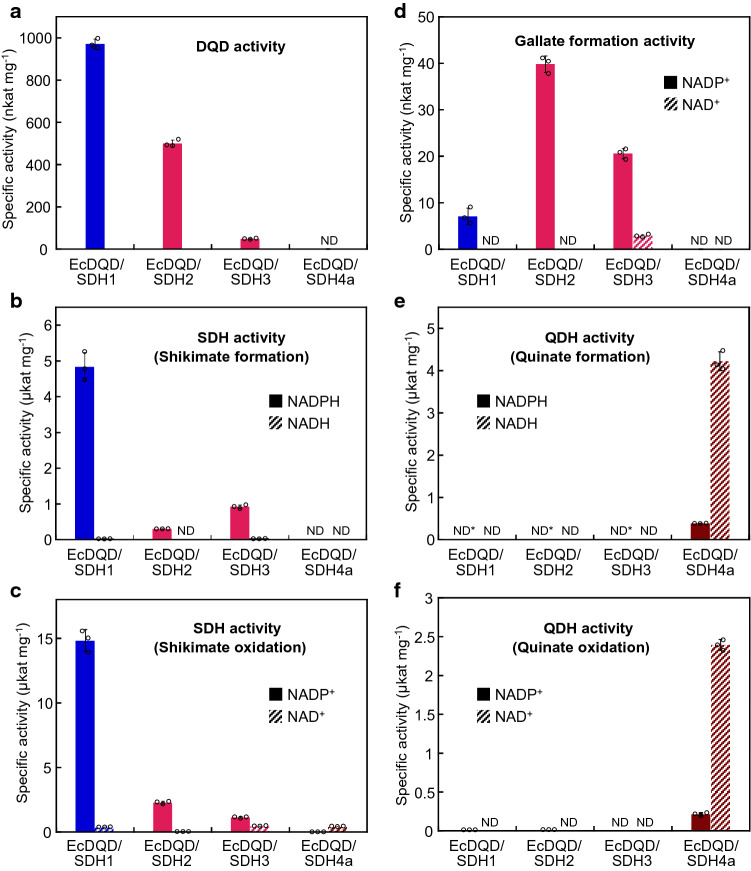
Enzymatic activities of recombinant EcDQD/SDHs. Recombinant proteins were assayed at 30 °C in 4 mM 3-DHQ at pH 7 (**a**), 12 mM 3-DHS and 0.4 mM NAD(P)H at pH 6.5 (**b**), 12 mM shikimate and 2 mM NAD(P)^+^ at pH 9 (**c**), 30 mM 3-DHS and 6 mM NAD(P)^+^ at pH 10.5 (**d**), 8 mM 3-DHQ and 0.4 mM NAD(P)H at pH 7.5 (**e**), or 6 mM quinate and 1 mM NAD(P)^+^ at pH 9 (**f**). Enzymatic activities were determined by measuring the production of 3-DHS (**a**) or gallate (**d**) by HPLC or by monitoring the consumption (**b**, **e**) or production (**c**, **f**) of NAD(P)H spectrophotometrically. e*Activity was determined by measuring the quinate production by HPLC and not by monitoring NADPH consumption because the reactions from 3-DHQ to shikimate through 3-DHS also consume NADPH. Data are presented as the mean ± SD (*n* = 3). Activities less than 10 nkat mg^−1^ (**a**, **b**, **e**) or 1 nkat mg^−1^ (**c**, **d**, **f**) are indicated as “not detected (ND)”

First, we assessed the enzymatic reaction specificity and cofactor preference of the four EcDQD/SDHs in assays with different substrate and cofactor combinations (Fig. 3). The DQD activity was observed for EcDQD/SDH1, 2, and 3, but not for EcDQD/SDH4a (Fig. 3a). Among the active enzymes, EcDQD/SDH1 exhibited the highest DQD activity, followed by EcDQD/SDH2 (about 50% of the EcDQD/SDH1 activity) and EcDQD/SDH3 (about 5% of the EcDQD/SDH1 activity).

To assess the SDH activity, shikimate formation from 3-DHS as well as shikimate oxidation to 3-DHS were analyzed (Fig. 3b, c). For both reactions, measurable catalytic activities were detected for EcDQD/SDH1–3, but the activities of EcDQD/SDH2 and 3 were less than 20% of those of EcDQD/SDH1. Regarding the cofactor, EcDQD/SDH1–3 had a clear preference for NADPH/NADP^+^ over NADH/NAD^+^. In contrast, EcDQD/SDH4a lacked shikimate formation activity. For the reverse reaction, the conversion of shikimate to 3-DHS, EcDQD/SDH4a displayed low enzymatic activity with a preference for NAD^+^ as the cofactor (Fig. 3c).

Both EcDQD/SDH2 and 3 exhibited relatively high gallate formation activity, in contrast to the low activity of EcDQD/SDH1 (Fig. 3d). The preferred cofactor in this reaction was NADP^+^.

The reversible quinate formation from 3-DHQ was catalyzed only by EcDQD/SDH4a (Fig. 3e and f), with NADH/NAD^+^ as the preferred cofactor. The reaction specificity of EcDQD/SDH4a confirmed the sequence-based prediction that EcDQD/SDH4a is a functional QDH enzyme. Hence, we propose that this enzyme should be renamed EcQDHa and its closest relative, EcDQD/SDH4b, should be renamed EcQDHb.

### Effects of pH and divalent metal ions on enzymatic activities of EcDQD/SDHs

**Fig. 4:**
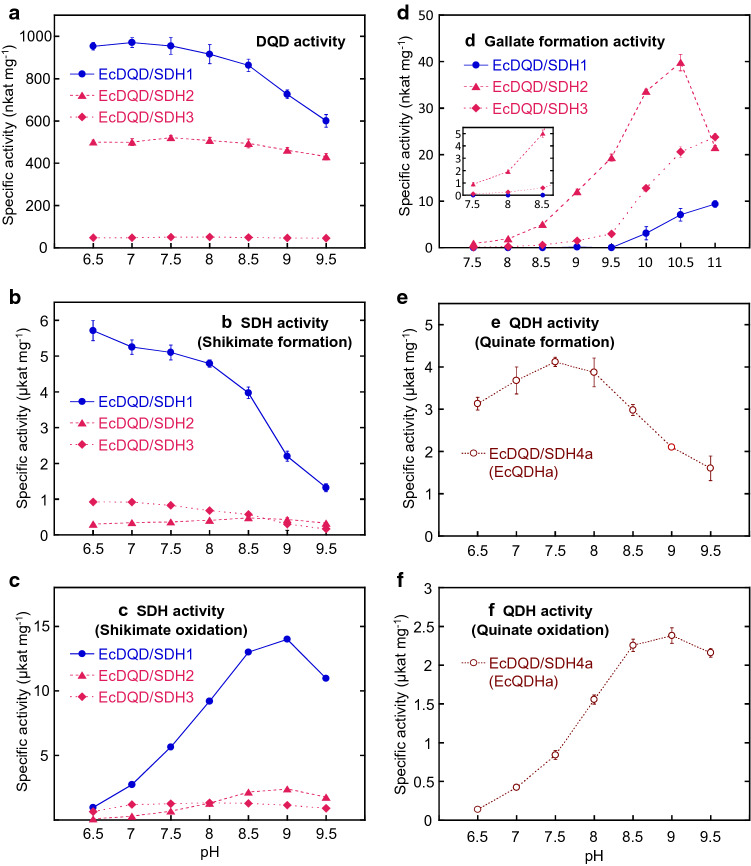
Effect of pH on the enzyme activities of recombinant EcDQD/SDHs. Recombinant proteins were assayed at 30 °C in 4 mM 3-DHQ (**a**), 12 mM 3-DHS and 0.4 mM NADPH (**b**), 12 mM shikimate and 2 mM NADP^+^ (**c**), 30 mM 3-DHS and 6 mM NADP^+^ (**d**), 8 mM 3-DHQ and 0.4 mM NADH (**e**), or 6 mM quinate and 1 mM NAD^+^ (**f**). Enzymatic activities were determined by measuring the production of 3-DHS (**a**) or gallate (**d**) by HPLC or by monitoring the consumption or production of NADPH (**b**, **c**) or NADH (**e**, **f**) spectrophotometrically. Data are presented as the mean ± SD (*n* = 3)

The effects of pH on enzyme activities were examined using active EcDQD/SDHs and the preferred cofactor for each reaction. The DQD activity was associated with the broadest optimal pH range (Fig. 4a). Specifically, EcDQD/SDH2 and 3 maintained their DQD activity from mildly acidic to relatively basic conditions (pH 9 and 9.5). Oxidation reactions (i.e., shikimate and quinate oxidation as well as gallate formation) had a higher optimal pH (pH 9–10.5; Fig. 4c, d, and f) than reduction reactions (i.e., shikimate and quinate formation) (pH 6.5–7.5; Fig. 4b and e). An exception to this trend was the shikimate formation catalyzed by EcDQD/SDH2, which had an optimal pH of 8.5 (slightly basic conditions). The optimal pH for gallate formation was especially high (pH 10.5 or higher; Fig. 4d).

Some SDHs are reportedly activated by divalent metal ions, including Ca^2+^ and Mg^2+^ (Schmidt et al. 1991; Díaz and Merino 1997). Thus, we evaluated the effects of divalent metal ions on the gallate formation activity of EcDQD/SDH2 and 3. None of the tested metal ions activated the gallate formation activity of these two enzymes (Table S4). Moreover, Cu^2+^, Mn^2+^, Zn^2+^, and Co^2+^ inhibited gallate formation.

### Kinetic parameters

The active EcDQD/SDHs displayed Michaelis–Menten behaviors for the investigated reactions (Fig. S5). Kinetic parameters were determined at the optimal pH in the presence of the preferred cofactor for each active EcDQD/SDH and reaction (Table 1).

**Table 1:** Kinetic parameters of recombinant EcDQD/SDHs

Reaction (Substrate → Product) [Cofactor]	Variable molecule	Enzyme	pH	*K*_m_ (μM)	*V*_max_ (nkat mg^−1^)	*k*_cat_ (s^−1^)	*k*_cat_/*K*_m_ (mM^−1 s−1^)
DQD activity							
(3-DHQ → 3-DHS) [None]	3-DHQ	EcDQD/SDH1	7	449 ± 22	1050 ± 10	60.4 ± 0.7	135
		EcDQD/SDH2	7	322 ± 13	540 ± 5	30.9 ± 0.3	96.2
		EcDQD/SDH3	7	882 ± 42	63.9 ± 0.9	3.67 ± 0.05	4.16
SDH activity							
Shikimate formation (3-DHS → Shikimate) [NADPH]	3-DHS	EcDQD/SDH1	6.5	381 ± 34	5300 ± 80	307 ± 5	805
		EcDQD/SDH2	8.5	8830 ± 1210	821 ± 57	47.0 ± 3.3	5.32
		EcDQD/SDH3	6.5	878 ± 106	1000 ± 30	57.6 ± 1.7	65.6
	NADPH	EcDQD/SDH1	6.5	49.5 ± 3.6	6730 ± 150	389 ± 9	7860
		EcDQD/SDH2	8.5	4.38 ± 0.43	482 ± 8	27.6 ± 0.5	6300
		EcDQD/SDH3	6.5	23.1 ± 1.4	1290 ± 20	74.1 ± 1.2	3210
Shikimate oxidation (Shikimate → 3-DHS) [NADP^+^]	Shikimate	EcDQD/SDH1	9	32.6 ± 1.9	13,000 ± 100	755 ± 6	23,200
		EcDQD/SDH2	9	2050 ± 200	2550 ± 70	146 ± 4	71.3
		EcDQD/SDH3	9	3640 ± 360	1400 ± 50	80.7 ± 2.9	22.2
	NADP^+^	EcDQD/SDH1	9	56.5 ± 2.3	13,700 ± 100	792 ± 6	14,000
		EcDQD/SDH2	9	65.2 ± 2.7	2350 ± 20	135 ± 1	2070
		EcDQD/SDH3	9	41.5 ± 2.1	1220 ± 10	70.2 ± 0.6	1690
Gallate formation activity							
(3-DHS → Gallate) [NADP^+^]	3-DHS	EcDQD/SDH2	10.5	9440 ± 780	50.1 ± 1.3	2.87 ± 0.07	0.304
		EcDQD/SDH3	10.5	30,600 ± 2700	37.2 ± 1.6	2.14 ± 0.09	0.0700
QDH activity							
Quinate formation (3-DHQ → Quinate) [NADH]	3-DHQ	EcDQD/SDH4a (EcQDHa)	7.5	1400 ± 120	5000 ± 130	281 ± 7	201
	NADH	EcDQD/SDH4a (EcQDHa)	7.5	102 ± 9	5300 ± 150	298 ± 8	2920
Quinate oxidation (Quinate → 3-DHQ) [NAD^+^]	Quinate	EcDQD/SDH4a (EcQDHa)	9	164 ± 10	2250 ± 30	127 ± 2	771
	NAD^+^	EcDQD/SDH4a (EcQDHa)	9	53.7 ± 1.7	2470 ± 20	139 ± 1	2590

Purified recombinant proteins were assayed with at least eight substrate or cofactor concentrations at the optimal pH for each reaction at 30 °C. Kinetic profiles are presented in Fig. S5. Data are presented as estimates ± SEs (*n* = 32–44)

Among the enzymes under investigation, EcDQD/SDH1 showed the highest catalytic efficiencies (*k*_cat_/*K*_m_) for DQD and SDH activities. Additionally, EcDQD/SDH2 exhibited a lower, but still comparable, catalytic efficiency for the DQD activity and low efficiency for the SDH activity. The EcDQD/SDH3 enzyme exhibited inefficient DQD and SDH activities. The cofactor kinetics for NADPH and NADP^+^ roughly reflected the kinetic behavior of EcDQD/SDH1–3 for substrates 3-DHS and shikimate, respectively (Table 1).

For the production of gallate from 3-DHS, kinetic constants were measurable exclusively for EcDQD/SDH2 and 3. The catalytic efficiencies of gallate formation were extremely low. The *K*_m_ values for 3-DHS were in the mM range, implying poor substrate affinity, and were accompanied by low reaction velocities (*V*_max_; Table 1).

### Gene expression and metabolite concentration

**Fig. 5:**
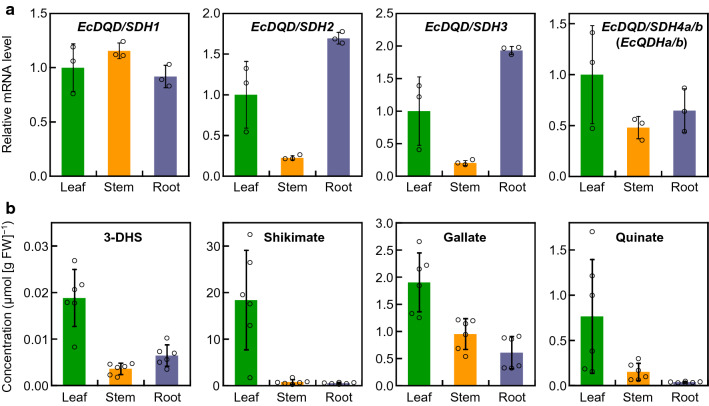
Expression of *EcDQD/SDH* genes (**a**) and concentrations of DQD/SDH substrates and products (**b**) in *Eucalyptus camaldulensis* organs. **a** Relative mRNA levels were determined in the leaves, stems, and roots under normal growth conditions. The *EcDQD/SDH4a* and *EcDQD/SDH4b* expression levels were quantified as the total mRNA abundance for sequence variants *a* and *b*. The mRNA level in leaves was defined as 1. The *EcActin* gene served as an internal control. Data are presented as the mean ± SD (*n* = 3). **b** Metabolite concentrations were determined in the leaves, stems, and roots under normal growth conditions. Data are presented as the mean ± SD (*n* = 6)

The expression of *EcDQD/SDH* genes in *E. camaldulensis* cultivated under normal growth conditions was investigated by quantitative real-time RT-PCR. All four analyzed *EcDQD/SDH* genes were expressed in the leaves, stems, and roots (Fig. 5a). The substrates and products of the DQD/SDH-catalyzed reactions in *E. camaldulensis* were quantified by GC–MS. Specifically, 3-DHS, shikimate, gallate, and quinate were detected in the leaves, stems, and roots (Fig. 5b), with the highest concentrations detected in the leaves (*P* < 0.05, Tukey–Kramer test). Although 3-DHQ was also detected in *E. camaldulensis*, its concentration was too low to be quantified.

**Fig. 6:**
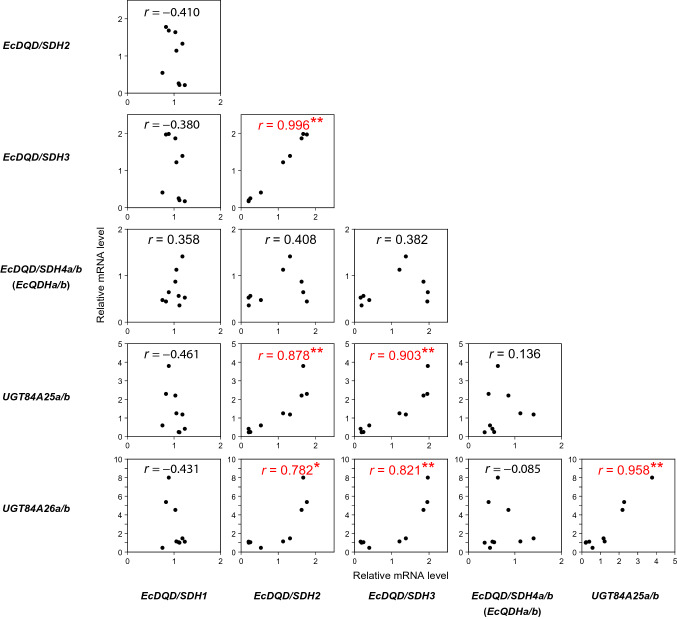
Comparison of *EcDQD/SDH*, *UGT84A25a/b*, and *UGT84A26a/b* expression patterns in *Eucalyptus camaldulensis*. Relative *EcDQD/SDH* mRNA levels in the leaves, stems, and roots (Fig. 5a) were plotted against the relative *UGT84A25a/b* and *UGT84A26a/b* mRNA levels in the same samples. The mean mRNA level in leaves was defined as 1. The *EcActin* gene served as an internal control. Asterisks indicate a significant correlation between two genes at *P < 0.05 and **P < 0.01 (Pearson’s correlation test; *n* = 9)

A comparison of gene expression patterns revealed a correlation between the *EcDQD/SDH2* and *EcDQD/SDH3* expression levels as well as between the expression of these two genes and the expression of *UGT84A25a/b* and *UGT84A26a/b* (Fig. 6), which catalyze the step after gallate formation in the hydrolyzable tannin biosynthetic pathway (i.e., β-glucogallin formation from gallate and UDP-glucose) (Fig. 1) (Tahara et al. 2018). The co-expression of these four genes suggests that the encoded enzymes are associated with the same metabolic pathway.

### Effects of Al on EcDQD/SDHs

**Fig. 7:**
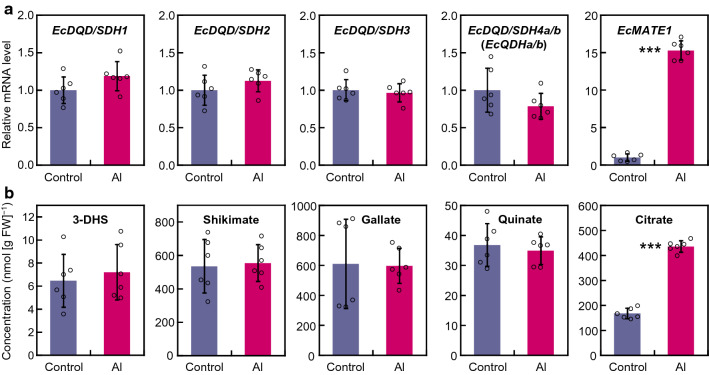
Effect of aluminum on *EcDQD/SDH* gene expression (**a**) and the concentrations of DQD/SDH substrates and products in roots of *Eucalyptus camaldulensis* (**b**). Relative mRNA levels and metabolite concentrations were determined after a 5-day exposure to 0 or 1.5 mM AlCl_3_ in nutrient solution (pH 4). The *EcDQD/SDH4a* and *EcDQD/SDH4b* expression levels were quantified as the total mRNA abundance for sequence variants *a* and *b*. The mRNA level in the control roots was defined as 1. The mRNA level of the aluminum-responsive gene *EcMATE1* (accession number AB725912) was used as a positive control for the aluminum treatment. The *EcActin* gene served as an internal control. The citrate concentration was used as a positive control for the aluminum treatment. Data are presented as the mean ± SD (*n* = 6). Asterisks indicate a significant difference between treatments at ***P < 0.001 (Student’s *t* test)

We previously determined that the concentration of oenothein B, a hydrolyzable tannin, increases in *E. camaldulensis* roots in response to Al stress (Tahara et al. 2014). Thus, we investigated the effects of Al-stress conditions on *EcDQD/SDH* gene expression and substrate and product concentrations in *E. camaldulensis*. The long-term (5 days; Fig. 7a) and short-term (24 h; Fig. S6) Al treatments did not upregulate *EcDQD/SDH* expression levels. This is in contrast to the effects of Al stress on the expression of *EcMATE*, a confirmed Al-induced gene (Sawaki et al. 2013).

The Al treatment did not appear to affect the 3-DHS, shikimate, and quinate concentrations in the roots, whereas it increased the citrate concentration (Fig. 7b). A previous study showed that citrate accumulates in response to Al stress (Tahara et al. 2008). The constitutive expression of *EcDQD/SDH2* and *3* and the 3-DHS and gallate contents regardless of Al stress suggest that the Al-induced accumulation of oenothein B is not due to regulated gallate formation.

## Discussion

### EcDQD/SDH2 and 3 are involved in gallate formation

Both EcDQD/SDH2 and 3 exhibited three catalytic activities: gallate formation, DQD, and SDH activities (Fig. 3a–d). Although gallate-forming DQD/SDHs have been detected in two other plant species, *J. regia* and *V. vinifera*, precise kinetic data for gallate formation are not available, most likely because of the observed low catalytic activities (Muir et al. 2011; Bontpart et al. 2016). In these previous studies, only kinetic data for the SDH activity were reported. Precisely quantifying enzymatic gallate formation is challenging because of the substantial non-enzymatic background production of gallate under alkaline assay conditions. In this study, we generated kinetic data for enzymatic gallate formation for the first time (Table 1; Figs. 3 and 4) by carefully eliminating the non-enzymatic background (see “Material and methods”). The kinetic constants revealed that EcDQD/SDH2 and 3 are relatively inefficient enzymes regarding all three catalytic activities. Gallate formation was characterized by particularly low substrate affinities and slow reaction velocities (Table 1). These observations may be typical features of enzymes involved in the formation of natural products that accumulate over long periods in long-lived plant species. However, in terms of evolution, our findings may suggest that these enzymes are undergoing a neofunctionalization from the “classical” SDH activity to the gallate formation activity and are currently in an intermediate stage, wherein they still exhibit both activities, but with low efficiencies.

This study revealed the first examples of plant gallate-forming DQD/SDHs maintaining DQD activity (Table 1; Fig. 3). The crystal structure of AtDQD/SDH indicated that the DQD and SDH active sites are in close proximity and face each other (Singh and Christendat 2006), thereby suggesting a direct route for transferring the intermediate 3-DHS from the DQD active site to the SDH (i.e., gallate formation) active site in EcDQD/SDH2 and 3. Hence, our kinetic data imply that the DQD activity, as well as the shikimate oxidation activity of EcDQD/SDH2 and 3, can provide 3-DHS to overcome the low substrate affinity of the gallate formation activity. Taken together, our results support the idea that EcDQD/SDH2 and 3 produce gallate *in planta* by two metabolic pathways: (1) from 3-DHQ via 3-DHS using DQD and gallate formation activities and (2) from shikimate via 3-DHS using shikimate oxidation and gallate formation activities.

We used the in vitro assay conditions established in this study to systematically investigate the pH dependence of the gallate formation activity of recombinant EcDQD/SDH2 and 3. Our results indicated the optimal pH was 10.5 for EcDQD/SDH2 and even higher for EcDQD/SDH3 (Fig. 4d). To date, the pH optima for enzymatic gallate formation by recombinant SDHs have not been reported. Bontpart et al. (2016) used pH 9.0 for assaying SDH enzymes from *Vitis* species. This, at least, supports our findings that the SDH-mediated gallate formation activity requires alkaline conditions and peaks at an unphysiologically high pH when assayed in vitro. An unphysiologically high pH optimum (pH 10) was also reported for a partially purified enzyme from *Betula pubescens* leaves which catalyzes the NADP^+^-dependent conversion of 3-DHS to gallate (Ossipov et al. 2003). In buffer systems with the pH maintained between 7.5 and 8.5, which is close to that under physiological conditions, EcDQD/SDH2 and 3 exhibited low but measurable gallate formation activity (Fig. 4d). In silico predictions suggested that EcDQD/SDH2 and 3 are localized to the plastids, which are characterized by alkaline conditions (pH close to 8.0) (Song et al. 2004). This implies that *in planta*, EcDQD/SDH2 and 3 catalyze gallate formation from the shikimate pathway intermediate 3-DHS under sub-optimal conditions. The kinetic data and the activity levels indicate that EcDQD/SDH2 may produce gallate more efficiently than EcDQD/SDH3 *in planta*.

In *E. camaldulensis*, the genes encoding EcDQD/SDH2 and 3 were most highly expressed in the leaves and roots, where hydrolyzable tannins accumulate (Fig. 5a). Unlike the other *EcDQD/SDH* genes under investigation, *EcDQD/SDH2* and *3* were co-expressed with *UGT84A25a/b* and *UGT84A26a/b* (Fig. 6), which encode the entry enzymes for hydrolyzable tannin biosynthesis (Tahara et al. 2018). This further supports the predicted role for EcDQD/SDH2 and 3 in gallate biosynthesis.

### EcDQD/SDH1 is a “classical” shikimate pathway enzyme

In the current study, EcDQD/SDH1 exhibited substantial DQD and SDH activities and minor gallate formation activity (Fig. 3a–d). The catalytic efficiency (*k*_cat_/*K*_m_) of EcDQD/SDH1 for shikimate formation from 3-DHS was considerably higher than that for DQD activity (Table 1). This enables the enzyme to channel the metabolite 3-DHQ via 3-DHS to shikimate. In *E. camaldulensis*, *EcDQD/SDH1* was expressed at similar levels in different plant organs (Fig. 5a). This is consistent with a role for the enzyme in the essential shikimate pathway. The gallate formation activity of EcDQD/SDH1 reflects certain plasticity at the SDH active site. However, compared with the DQD and SDH activities, gallate production should be considered as a catalytic side activity of EcDQD/SDH1. In *E. camaldulensis*, *EcDQD/SDH1* was not co-expressed with *UGT84A25a/b* and *UGT84A26a/b* (Fig. 6). Together with the poor gallate formation activity, this finding implies EcDQD/SDH1 lacks a physiological role in gallate metabolism.

### EcQDHa/b might be involved in quinate catabolism

**Fig. 8:**
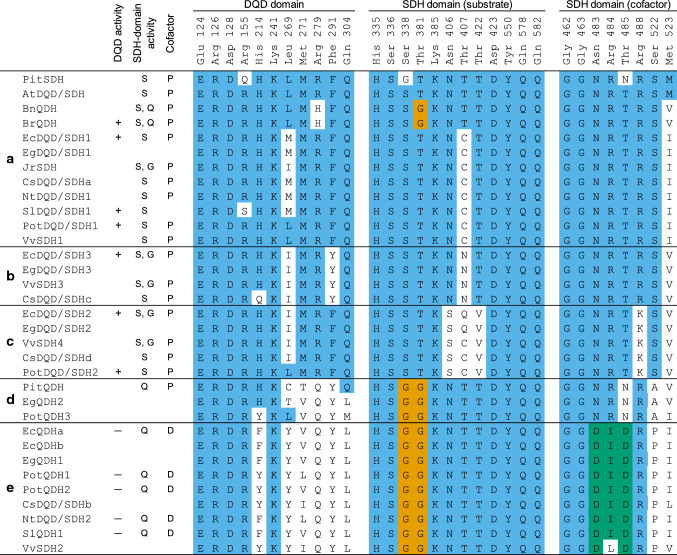
Comparison of key active site residues in EcDQD/SDHs and other functionally characterized DQD/SDH family members in seed plants. Key active site residues involved in catalysis and binding of substrates and cofactors (Singh and Christendat 2006, 2007) were identified after DQD/SDH amino acid sequences (Fig. 2) were aligned with MUSCLE. Amino acid positions are based on AtDQD/SDH. Significant DQD-domain activity is indicated by “ + ”, whereas trace or undetectable DQD activity is indicated by “ − ”. The main SDH-domain activity is indicated by “S” (SDH) or “Q” (QDH). The SDH domain exhibiting gallate formation activity is indicated by “G”. The cofactor preference of the SDH domain is indicated by “P” (NADP) or “D” (NAD). Blank indicates not tested. Key residues identical to AtDQD/SDH are highlighted in blue. Amino acid substitutions conferring QDH activity are in orange. An aspartate-isoleucine-aspartate (DID) sequence motif indicative of NAD preference is outlined in green. References are listed in Table S3

In silico predictions revealed EcQDHa/b (initially named as EcDQD/SDH4a/b) are localized outside of plastids. This is in accordance with the suggestion that NAD-dependent QDHs are cytoplasmic enzymes (Ding et al. 2007; Gritsunov et al. 2018). Regarding function, the authors argued that non-plastidial NAD-dependent QDHs are involved in the catabolism of quinate to produce 3-DHQ, whereas the biosynthesis of quinate from the shikimate pathway intermediate 3-DHQ is catalyzed by plastid-localized NADP-dependent QDHs, which form another clade within the QDH enzyme family (group D in Fig. 2) (Carrington et al. 2018; Gritsunov et al. 2018). Interestingly, an analysis of the *Eucalyptus grandis* (Eg) genome sequence uncovered two candidate genes predicted to encode QDH enzymes, *EgQDH1*, which is an ortholog of *EcQDHa/b*, and *EgQDH2* (Fig. 2). The protein encoded by *EgQDH2* carries the Asn-Arg-Asn (NRN) peptide sequence motif (Fig. 8), which is indicative of a preference for NADP as a cofactor (Peek and Christendat 2015). Moreover, in silico predictions suggested that EgQDH2 is an enzyme that is localized in plastids. Accordingly, *EgQDH2* likely encodes a plastid-localized NADP-dependent QDH involved in quinate biosynthesis in *E. grandis*. Unlike the available *E. grandis* genome sequence, the published *E. camaldulensis* genome sequence is still a draft version. Thus, for quinate biosynthesis in *E. camaldulensis*, we predict the existence of a second plastidial NADP-dependent QDH (i.e., ortholog of EgQDH2) that remains to be identified. The EcQDHa/b identified in the current study are most likely involved in the branching off of quinate from the chlorogenate biosynthetic pathway and in utilizing the quinate derived from chlorogenate degradation in older plant tissues.

### Molecular features governing the gallate formation activity of EcDQD/SDH2 and 3

**Fig. 9:**
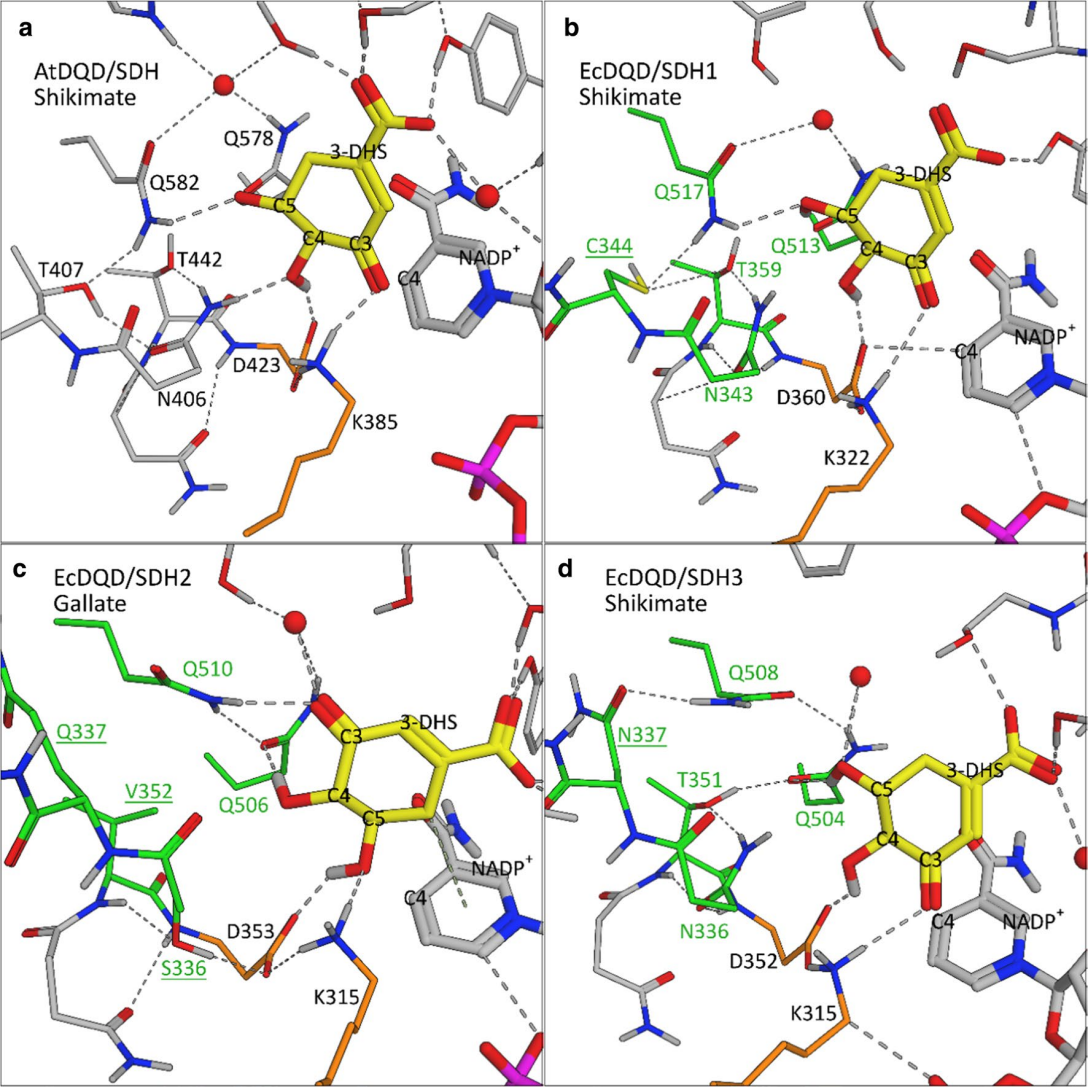
Model structures of the SDH ternary complex of AtDQD/SDH (**a**) and EcDQD/SDH1–3 (**b, c, d**). Key substrate-binding residues are in gray (AtDQD/SDH) or green (EcDQD/SDHs). Substituted amino acids are underlined. The catalytic dyad is marked in orange. The substrate 3-DHS (yellow, bold) is presented in the orientation required for shikimate (**a**, **b**, **d**) or gallate (**c**) formation. Water molecules are represented by red spheres. Hydrogen bridges are indicated as dashed lines

Regarding the “classical” DQD/SDH enzyme AtDQD/SDH, crystal structure and mutant analyses unraveled the catalytic center and the hydrogen bond network for substrate binding (Singh and Christendat 2006, 2007). To elucidate the molecular mechanisms underlying the gallate formation activity, we generated structural models for EcDQD/SHD1–3 with AtDQD/SDH as the template (Fig. 9).

The EcDQD/SDH structural models indicate that the functional change from SDH to gallate formation activity is mainly driven by a shift of the 3-DHS orientation in the enzyme–substrate complex. Gallate-forming EcDQD/SDH2 and 3 enzymes carry amino acid substitutions corresponding to Asn406, Thr407, and Thr422 in AtDQD/SDH (Fig. 8). These amino acids form part of the hydrogen bond network required for placing the C3 keto group of 3-DHS at a favorable distance to the catalytic dyad (Lys385 and Asp423) to facilitate the H transfer for shikimate formation (Peek and Christendat 2015; Fig. 9a). In EcDQD/SDH1 (Fig. 9b), this interaction is maintained because the thiol group of Cys344 can substitute for the hydroxyl group of Thr407 in AtDQD/SDH. In EcDQD/SDH2, however, the hydrogen bond network exhibits a pronounced adjustment (Fig. 9c). Most obviously, Ser336 does not interact with the substrate 3-DHS, in contrast to the corresponding Asn406 in AtDQD/SDH. Additionally, the non-polar Val352 might turn the hydroxyl group of Ser336 away from the active site and toward the protein backbone, thereby further diminishing a potential interaction with the substrate. Unlike the H-bonded Thr407–Gln582 in AtDQD/SDH, the corresponding Gln337 and Gln510 cannot interact, most likely because of the bulky side chain of glutamine. Consequently, the amide group of EcDQD/SDH2 Gln510 turns by nearly 90° relative to Gln582 in AtDQD/SDH, exposing the amide nitrogen to interact with the C3 keto group of 3-DHS. This locks the substrate 3-DHS into a specific position that supports the proton abstraction from C5 leading to gallate formation.

In EcDQD/SDH3 (Fig. 9d), the adjustment of the hydrogen bond network is less pronounced than that in EcDQD/SDH2. Specifically, the keto group of Asn337 interacts with Gln508, leading to a slight twist of the Gln508 amide group, but in the opposite direction than that observed for the corresponding Gln510 in EcDQD/SDH2 (Fig. 9c, d). This might allow 3-DHS to adopt an appropriate orientation for the production of both shikimate and gallate, but with a higher specificity toward shikimate formation.

### Amino acid substitutions define the QDH activity of EcQDHa

Recent studies confirmed that plant QDH enzymes evolved from DQD/SDH precursors by neofunctionalization and loss of DQD activity, followed by their differentiation into NADP-dependent and NAD-dependent clades (Carrington et al. 2018; Gritsunov et al. 2018). The authors assigned specific amino acid substitutions to these evolutionary changes. For example, a single amino acid substitution (Thr381Gly) was sufficient for acquiring the QDH activity from SDH (Carrington et al. 2018; Gritsunov et al. 2018). An additional Ser338Gly substitution enhanced the efficiency of the QDH activity (Carrington et al. 2018). Sequence analyses indicated that EcQDHa/b (EcDQD/SDH4a/b) carry the Thr381Gly and Ser338Gly substitutions (Fig. 8), which may explain the acquisition of QDH activity and the loss of SDH activity (Fig. 3b and c).

Regarding the cofactor preferences of DQD/SDH and QDH enzymes, a sequence motif at position 483–485 may be essential. Accordingly, the Asn-Arg-Thr (NRT) motif is associated with a preference for NADPH/NADP^+^, whereas the Asp-Ile-Asp (DID) sequence motif is correlated with a preference for NADH/NAD^+^ (Peek and Christendat 2015; Gritsunov et al. 2018). Our assay data revealing the NADH/NAD^+^ specificity for EcQDHa and the NADPH/NADP^+^ specificity for EcDQD/SDH1–3 were consistent with the predictions based on the sequence motifs (Figs. 3 and 8).

The loss of DQD activity by QDH enzymes might be required to prevent the possible competition between two active sites for the substrate 3-DHQ (Gritsunov et al. 2018). Sequence analyses resulted in the identification of essential amino acid residues within the DQD domain (Singh and Christendat 2006), which have been substituted in the non-functional DQD domains of QDH enzymes. In EcQDHa/b, we determined that a catalytic amino acid (His214Phe) and a key substrate-binding region (Arg279Gln) were affected by substitutions (Fig. 8), which may explain the loss of DQD activity.

## Conclusion

In this study, our examination of the Al-tolerant tree species *E. camaldulensis* uncovered five genes encoding DQD/SDH family members (Fig. S7). Both EcDQD/SDH2 and 3 exhibit distinct gallate formation activities. These two are the first enzymes whose kinetic constants and pH optima for gallate formation have been determined. The gallate formation activity is correlated with specific amino acid substitutions in the substrate-binding pocket of the enzymes. Additionally, EcDQD/SDH2 and 3 were also revealed to combine DQD and shikimate oxidation activities, which can provide the substrate 3-DHS for gallate formation. Both EcDQD/SDH2 and 3 are co-expressed with enzymes from the hydrolyzable tannin biosynthetic pathway and are most likely involved in the formation of gallate *in planta*. Moreover, EcDQD/SDH1 combines the DQD activity with the “classical” SDH activity and minimal gallate formation activity. This enzyme is predicted to catalyze the third and fourth steps of the shikimate pathway. The *EcQDHa* and *EcQDHb* (*EcDQD/SDH4a* and *EcDQD/SDH4b*) genes represent allelic variants of a gene encoding a functional QDH enzyme. Specific amino acid substitutions explain the acquisition of QDH activity and the loss of DQD and SDH activities in these enzymes. The genes encoding EcDQD/SDH2 and 3 are candidates for manipulating gallate production in *E. camaldulensis*, with possible implications for the Al tolerance of this tree species.

### Author contributions statement

KT cloned EcDQD/SDH-cDNAs, produced the recombinant enzymes, and performed all enzymatic assays, including enzyme-kinetic calculations and GC–MS-based compound verification. He performed the phylogenetic analysis of plant DQD/SDH family enzymes and did the quantitative real-time RT-PCR in *E. camaldulensis*. He was involved in the conceptualization of the project, in writing and editing of the manuscript, and in funding acquisition. MN was involved in genome-based EcDQD/SDH-cDNA identification, development of cDNA cloning strategies and in phylogenetic analyses. EF developed the EcDQD/SDH structure models, designed Fig. 9 and interpreted the model structures with regard to EcDQD/SDH reaction mechanisms and evolutionary neofunctionalization. SM extracted and derivatized metabolites for GC–MS analyses. TM conducted GC–MS analyses. CM was involved in project design, conceptualization, and planning of experimental strategies. He wrote parts of the discussion, contributed to final manuscript editing, and raised project funding. All authors read and approved the manuscript.

## Supplementary Information

Below is the link to the electronic supplementary material.

Supplementary file1 (DOCX 1917 KB)

## Abbreviations

3-DHQ: 3-Dehydroquinate
3-DHS: 3-Dehydroshikimate
DQD: Dehydroquinate dehydratase
GST: Glutathione *S*-transferase
QDH: Quinate dehydrogenase
SDH: Shikimate dehydrogenase
UGT: UDP glycosyltransferase
