# Supplementary material for: Dehydroquinate dehydratase/shikimate dehydrogenases involved in gallate biosynthesis of the aluminum-tolerant tree species Eucalyptus camaldulensis
Source: Planta. 2020 Dec 21;253(1):3. doi: 10.1007/s00425-020-03516-w (PMC7752791; doi:10.1007/s00425-020-03516-w)
Supplement: Supplementary file 1 — Supplementary file1 (DOCX 1917 KB) [file 425_2020_3516_MOESM1_ESM.docx]

**Planta**

**Dehydroquinate dehydratase/shikimate dehydrogenases involved in gallate biosynthesis of the aluminum-tolerant tree species *Eucalyptus camaldulensis***

**Ko Tahara, Mitsuru Nishiguchi, Evelyn Funke, Shin-Ichi Miyazawa, Takafumi Miyama, and Carsten Milkowski**

Corresponding author: Carsten Milkowski; Martin Luther University Halle-Wittenberg, AGRIPOLY: International Graduate School in Agricultural and Polymer Sciences, Betty-Heimann-Straße 3, D-06120 Halle, Germany; e-mail, carsten.milkowski@izn.uni-halle.de

Table S1 PCR primers used for cDNA cloning, subcloning, and quantitative real-time PCR analysis

Table S2 Standard assay conditions for testing EcDQD/SDH enzyme activities

Table S3 Accession numbers of DQD/SDHs and QDHs shown in Figs. 2 and 8

Table S4 Effect of divalent metal ions on gallate formation activities of EcDQD/SDH2 and 3

Fig. S1 Purification of recombinant GST-tagged EcDQD/SDH proteins expressed in *Escherichia coli*.

Fig. S2 Amino acid sequence alignment of EcDQD/SDHs and AtDQD/SDH.

Fig. S3 Detection of enzymatic activities of EcDQD/SDHs by HPLC.

Fig. S4 Validation of the enzymatic reaction products of EcDQD/SDHs by GC-MS.

Fig. S5 Michaelis-Menten kinetics of EcDQD/SDHs.

Fig. S6 Effect of a 24-h aluminum treatment on *EcDQD/SDH* gene expression in *Eucalyptus camaldulensis* roots.

Fig. S7 Proposed roles of EcDQD/SDH enzymes in gallate, shikimate, and quinate biosynthesis.

**Table S1** PCR primers used for cDNA cloning, subcloning, and quantitative real-time PCR analysis

| Purpose | Gene | Forward  or reverse | Sequence (5’ –3’) |
| --- | --- | --- | --- |
| cDNA cloning | *EcDQD/SDH1*  (EcC055014.20) | Fw  Rev | CGACGCTTCACCAACCTCC  GTTCAAGTTGGCTTGCGCTA |
|  | *EcDQD/SDH2*  (EcC035206.10) | Fw  Rev | CTCAGCGGAGGTAGCTTTAC  CCAATCTGATGCTTCATACCG |
|  | *EcDQD/SDH3*  (EcC054875.120) | Fw  Rev | ATCTCACCACACTCCAGCC  GATGGACGCCGCCCATCTA |
|  | *EcDQD/SDH4a/b*  (EcC015288.50) | Fw  Rev | AGCATTCTTGTTTCGGCACC  TTGCAGCATCGTCGTTGTTG |
|  |  |  |  |
| Subcloning | *EcDQD/SDH1* | Fw  Rev | TTGGATCCGCCTCCACTGGCAACGTC  AAGCGGCCGCTCAGTACTTCGACATAATTTTCCTG |
|  | *EcDQD/SDH2* | Fw  Rev | TTGGATCCACTCTCAGCAGCATCCCG  AAGCGGCCGCTCAACTGTTCTTCACCAATGTATCC |
|  | *EcDQD/SDH3* | Fw  Rev | TTGGATCCGGCAGCGTTCCGTTCACTAC  AAGCGGCCGCCTATGCATGTTTCTCCATGAGTGTC |
|  | *EcDQD/SDH4a* | Fw  Rev | TTGGATCCGGCAGCCTCTCCCTCTCC  AAGCGGCCGCTCAGAACTTGGCCAAAACTATCTCC |
|  |  |  |  |
| Real-time PCR | *EcDQD/SDH1* | Fw  Rev | CTAGTCTTTGATGCTGTGTACACC  CATATTGCCCATATGCCTGTCC |
|  | *EcDQD/SDH2* | Fw  Rev | TGCCATCTACACGCCAAAATTG  CAGTTGCTTTGGTGCAGGATAC |
|  | *EcDQD/SDH3* | Fw  Rev | ATGCCATTTACACACCAAAGGAC  GTCCTGAACAATTCCTCCGGG |
|  | *EcDQD/SDH4a/b* | Fw  Rev | GCCAGTTCAATCTCTTTACCGG  GAACTTGGCCAAAACTATCTCCC |
|  | *UGT84A25a/b* | Fw  Rev | CAAGTCAACGGCCAATGGC  CACCTCACGACACTACCTTCAG |
|  | *UGT84A26a/b* | Fw  Rev | CCTGGAGGTGCTGGCTGC  CACCTCACGACACCACCTTTAA |
|  | *EcMATE1*  (EcC054720.30) | Fw  Rev | AGTCTCCCTTATCAGCATTGCTTC  ACGTTGTGGAAGAAGTCCTTCTC |
|  | *EcActin*  (EcS556981.10) | Fw  Rev | GTTGCACCCCCTGAGAGAAAG  TTTCCTGTGGACGATGGATGG |

The added *Bam*HI and *Not*I restriction enzyme sites are underlined.

**Table S2** Standard assay conditions for testing EcDQD/SDH enzyme activities

| Reaction | Substrate | Cofactor | pH | Time | Product | Measurement |
| --- | --- | --- | --- | --- | --- | --- |
| DQD | 4 mM 3-DHQ | None | 7 | 2 min | 3-DHS | HPLC |
| Shikimate  formation | 12 mM 3-DHS | 0.4 mM NADPH  or NADH | 6.5 | 2 min | Shikimate | Spectrophotometry |
| Shikimate oxidation | 12 mM Shikimate | 2 mM NADP^+^  or NAD^+^ | 9 | 2 min | 3-DHS | Spectrophotometry |
| Gallate  formation^a^ | 30 mM 3-DHS | 6 mM NADP^+^  or NAD^+^ | 10.5 | 10 min | Gallate | HPLC |
| Quinate  formation | 8 mM 3-DHQ | 0.4 mM NADPH  or NADH | 7.5 | 2 min | Quinate | Spectrophotometry or HPLC |
| Quinate oxidation | 6 mM Quinate | 1 mM NADP^+^  or NAD^+^ | 9 | 2 min | 3-DHQ | Spectrophotometry |

All reactions were performed at 30 °C. DQD, dehydroquinate dehydratase; 3-DHQ, 3-dehydroquinate; 3-DHS, 3-dehydroshikimate. ^a^ 8 mM ascorbic acid was added to the reaction mixture to prevent gallate degradation

**Table S3** Accession numbers of DQD/SDHs and QDHs shown Figs. 2 and 8

| Species | Protein name | Accession | Reference |
| --- | --- | --- | --- |
| *Arabidopsis thaliana* (At) | DQD/SDH | AAS76684 | Singh and Christendat 2006  Gritsunov et al. 2018 |
| *Brassica napus* (Bn) | QDH | GSBRNA2T00027085001 | Gritsunov et al. 2018 |
| *Brassica rapa* (Br) | QDH | Brara.E03289.1 | Gritsunov et al. 2018 |
| *Camellia sinensis* (Cs) | DQD/SDHa | AYP64306 | Huang et al. 2019 |
| *Camellia sinensis* (Cs) | DQD/SDHb | AYP64307 | Huang et al. 2019 |
| *Camellia sinensis* (Cs) | DQD/SDHc | AYP64308 | Huang et al. 2019 |
| *Camellia sinensis* (Cs) | DQD/SDHd | AYP64309 | Huang et al. 2019 |
| *Eucalyptus camaldulensis* (Ec) | DQD/SDH1 | BBL52470 | This study |
| *Eucalyptus camaldulensis* (Ec) | DQD/SDH2 | BBL52471 | This study |
| *Eucalyptus camaldulensis* (Ec) | DQD/SDH3 | BBL52472 | This study |
| *Eucalyptus camaldulensis* (Ec) | DQD/SDH4a (QDHa) | BBL52473 | This study |
| *Eucalyptus camaldulensis* (Ec) | DQD/SDH4b (QDHb) | BBL52474 | This study |
| *Eucalyptus grandis* (Eg) | DQD/SDH1 | Eucgr.J00263.1 | Not characterized |
| *Eucalyptus grandis* (Eg) | DQD/SDH2 | Eucgr.H04428.1 | Not characterized |
| *Eucalyptus grandis* (Eg) | DQD/SDH3 | Eucgr.H04427.1 | Not characterized |
| *Eucalyptus grandis* (Eg) | QDH1 | Eucgr.B01770.1 | Not characterized |
| *Eucalyptus grandis* (Eg) | QDH2 | Eucgr.H01214.1 | Not characterized |
| *Juglans regia* (Jr) | SDH | AAW65140 | Muir et al. 2011 |
| *Nicotiana tabacum* (Nt) | DQD/SDH1 | AAS90325 | Ding et al. 2007 |
| *Nicotiana tabacum* (Nt) | DQD/SDH2 | AAS90324 | Ding et al. 2007  Gritsunov et al. 2018 |
| *Pinus taeda* (Pit) | SDH |  | Carrington et al. 2018 |
| *Pinus taeda* (Pit) | QDH |  | Carrington et al. 2018 |
| *Populus trichocarpa* (Pot) | DQD/SDH1 (Poptr1) | Potri.010G019000.2 | Guo et al. 2014 |
| *Populus trichocarpa* (Pot) | DQD/SDH2 (Poptr5) | Potri.013G029800.1 | Guo et al. 2014 |
| *Populus trichocarpa* (Pot) | QDH1 (Poptr2) | Potri.013G029900.2 | Guo et al. 2014 |
| *Populus trichocarpa* (Pot) | QDH2 (Poptr3) | Potri.005G043400.1 | Guo et al. 2014 |
| *Populus trichocarpa* (Pot) | QDH3 (Poptr4) | Potri.014G135500.3 | Guo et al. 2014 |
| *Solanum lycopersicum* (Sl) | DQD/SDH1 | AAC17991 | Bischoff et al. 2001 |
| *Solanum lycopersicum* (Sl) | QDH | Solyc10g038080.1.1 | Gritsunov et al. 2018 |
| *Vitis vinifera* (Vv) | SDH1 | KU163040 | Bontpart et al. 2016 |
| *Vitis vinifera* (Vv) | SDH2 | KU163041 | Bontpart et al. 2016 |
| *Vitis vinifera* (Vv) | SDH3 | KU163042 | Bontpart et al. 2016 |
| *Vitis vinifera* (At) | SDH4 | KU163043 | Bontpart et al. 2016 |

**Table S4** Effect of divalent metal ions on gallate formation activities of EcDQD/SDH2 and 3

| Compound | Relative activity (%) | |
| --- | --- | --- |
|  | EcDQD/SDH2 | EcDQD/SDH3 |
| None (control) | 100 ± 5 | 100 ± 16 |
| 5 mM EDTA-Na_2_ | 116 ± 5* | 84 ± 6 |
| 5 mM MgCl_2_ | 108 ± 5 | 99 ± 3 |
| 5 mM CaCl_2_ | 102 ± 4 | 101 ± 7 |
| 5 mM CoCl_2_ | 86 ± 6 | 73 ± 10** |
| 5 mM ZnCl_2_ | 72 ± 9** | 2 ± 4** |
| 5 mM MnCl_2_ | 66 ± 3** | 77 ± 7* |
| 5 mM CuCl_2_ | 15 ± 9** | 77 ± 9* |

Recombinant proteins were assayed in 10 mM 3-DHS and 6 mM NADPH^+^ at pH 10. Relative activity is expressed as the percentage of the control activity without the addition of metal ions. The control activity of EcDQD/SDH2 and 3 are 18.7 ± 1.0 and 4.7 ± 0.7 nkat mg^−1^, respectively. Data are presented as the mean ± SD (*n* = 3). Asterisks indicate significant differences between the control and each compound at **P* < 0.05 and ***P* < 0.01 (Dunnett’s test).

**Fig. S1** Purification of recombinant GST-tagged EcDQD/SDH proteins expressed in *Escherichia coli*. The crude protein extract (CE) and affinity-purified fraction (PF) were analyzed by SDS-PAGE and stained with Coomassie brilliant blue. The expected sizes for the recombinant EcDQD/SDHs after removal of the GST tag are 57.8 kDa (EcDQD/SDH1), 57.3 kDa (EcDQD/SDH2), 57.5 kDa (EcDQD/SDH3), and 56.2 kDa (EcDQD/SDH4a).

**Fig. S2** Amino acid sequence alignment of EcDQD/SDHs and AtDQD/SDH. The amino acid sequences were aligned with MUSCLE. Asterisks indicate the key active site residues of AtDQD/SDH involved in the binding and catalysis of a substrate (3-DHS or shikimate, red asterisks) and a cofactor [NADP(H), black asterisks] (Singh and Christendat 2006, 2007).

**Fig. S3** Detection of enzymatic activities of EcDQD/SDHs by HPLC. **a** To assay DQD activity, purified recombinant proteins (12.5 µg ml^−1^) were incubated in 5 mM 3-DHQ at pH 7 for 10 min at 30 °C. The reaction product, 3-DHS, in the assay mixtures was identified by comparing its retention time and absorption spectrum with those of an authentic standard.

**Fig. S3** (continued) **b** To assay shikimate formation activity, purified recombinant proteins (12.5 µg ml^−1^) were incubated in 5 mM 3-DHS and 5 mM NADPH at pH 6.5 for 10 min at 30 °C. Shikimate in the assay mixtures was identified by comparing its retention time and absorption spectrum with those of an authentic standard.

**Fig. S3** (continued) **c** To assay shikimate oxidation activity, purified recombinant proteins (1.25 µg ml^−1^) were incubated in 5 mM shikimate and 5 mM NADP^+^ at pH 9 for 10 min at 30 °C. The reaction product, 3-DHS, in the assay mixtures was identified by comparing its retention time and absorption spectrum with those of an authentic standard.

**Fig. S3** (continued) **d** To assay gallate formation activity, purified recombinant proteins (375 µg ml^−1^) were incubated in 5 mM 3-DHS and 5 mM NADP^+^ at pH 9.5 for 10 min at 30 °C. Gallate in the assay mixtures was identified by comparing its retention time and absorption spectrum with those of an authentic standard.

**Fig. S3** (continued) **e** To assay quinate formation activity, purified recombinant proteins (12.5 µg ml^−1^) were incubated in 5 mM 3-DHQ and 5 mM NADH at pH 7.5 for 10 min at 30 °C. Quinate in the assay mixtures was identified by comparing its retention time and absorption spectrum with those of an authentic standard.

**Fig. S3** (continued) **f** To assay quinate oxidation activity, purified recombinant proteins (12.5 µg ml^−1^) were incubated in 5 mM quinate and 5 mM NAD^+^ at pH 9 for 10 min at 30 °C. The reaction product, 3-DHQ, in the assay mixtures was identified by comparing its retention time and absorption spectrum with those of an authentic standard.

**Fig. S4** Validation of the enzymatic reaction products of EcDQD/SDHs by GC-MS. **a** Extracted-ion chromatograms of the authentic 3-DHS and the assay mixture (Fig. S3a) at *m*/*z* 417.2, corresponding to the molecular ion of 3-DHS, methoxyamine and tri(trimethylsilyl) derivative. A fragment ion of the substrate, 3-DHQ, was detected at 17.24 min. **b** Mass spectra of the authentic 3-DHS and the enzymatic reaction product at 16.25 min.

**Fig. S4** (continued) **c** Extracted-ion chromatograms of the authentic shikimate and the assay mixture (Fig. S3b) at *m*/*z* 462.2, corresponding to the molecular ion of shikimate, tetra(trimethylsilyl) derivative. **d** Mass spectra of the authentic shikimate and the enzymatic reaction product at 16.44 min.

**Fig. S4** (continued) **e** Extracted-ion chromatograms of the authentic 3-DHS and the assay mixture (Fig. S3c) at *m*/*z* 417.2, corresponding to the molecular ion of 3-DHS, methoxyamine and tri-TMS derivative. A fragment ion of the substrate, shikimate, was detected at 16.44 min. **f** Mass spectra of the authentic 3-DHS and the enzymatic reaction product at 16.25 min.

**Fig. S4** (continued) **g** Extracted-ion chromatograms of the authentic gallate and the assay mixture (Fig. S3d) at *m*/*z* 458.2, corresponding to the molecular ion of gallate, tetra(trimethylsilyl) derivative. **h** Mass spectra of the authentic gallate and the enzymatic reaction product at 17.98 min.

**Fig. S4** (continued) **i** Extracted-ion chromatograms of the authentic quinate and the assay mixture (Fig. S3e) at *m*/*z* 552.3, corresponding to the molecular ion of quinate, penta(trimethylsilyl) derivative. **j** Mass spectra of the authentic quinate and the enzymatic reaction product at 16.98 min.

**Fig. S4** (continued) **k** Extracted-ion chromatograms of the authentic 3-DHQ and the assay mixture (Fig. S3f) at *m*/*z* 507.2, corresponding to the molecular ion of 3-DHQ, methoxyamine and tetra(trimethylsilyl) derivative. **l** Mass spectra of the authentic 3-DHQ and the enzymatic reaction product at 17.24 min.

**Fig. S5** Michaelis-Menten kinetics of EcDQD/SDHs. **a** For the DQD reaction, recombinant proteins were assayed in 0.2–5 mM 3-DHQ at pH 7 and 30 °C. The assay was repeated four times.

**Fig. S5** (continued) For the shikimate formation reaction, recombinant proteins were assayed in 0.12–15 mM 3-DHS and 0.4 mM NADPH (**b**) or with 0.002–0.4 mM NADPH and 12 mM 3-DHS (**c**) at pH 6.5 (EcDQD/SDH1 and 3) or 8.5 (EcDQD/SDH2) at 30 °C. Each assay was repeated four times.

**Fig. S5** (continued) For the shikimate oxidation reaction, recombinant proteins were assayed in 0.12–15 mM shikimate and 2 mM NADP^+^ (**d**) or with 0.02–2.5 mM NADP^+^ and 12 mM shikimate (**e**) at pH 9 and 30 °C. Each assay was repeated four times.

**Fig. S5** (continued) **f** For the gallate formation reaction, recombinant proteins were assayed in 2–60 mM 3-DHS and 6 mM NADP^+^ at pH 10.5 and 30 °C. The assay was repeated four times.

**Fig. S5** (continued) For the quinate formation reaction, the recombinant protein was assayed in 0.12–10 mM 3-DHQ and 0.4 mM NADH (**g**) or 0.008–0.6 mM NADH and 8 mM 3-DHQ (**h**) at pH 7.5 and 30 °C. Each assay was repeated four times.

**Fig. S5** (continued) For the quinate oxidation reaction, the recombinant protein was assayed in 0.08–8 mM quinate and 1 mM NAD^+^ (**i**) or in 0.01–2 mM NAD^+^ and 6 mM quinate (**j**) at pH 9 and 30 °C. Each assay was repeated four times.

**Fig. S6** Effect of a 24-h aluminum treatment on *EcDQD/SDH* gene expression in *Eucalyptus camaldulensis* roots. Relative mRNA levels in the roots were determined after a 24-h treatment with 0 (control) or 1 mM AlCl_3_ in a calcium solution (pH 4). The *EcDQD/SDH4a* and *EcDQD/SDH4b* expression level was quantified as the total mRNA abundance for sequence variants *a* and *b*. The mRNA level in the control roots was defined as 1. The mRNA level of the aluminum-responsive gene *EcMATE1* (accession number AB725912) was used as a positive control for the aluminum treatment. The *EcActin* served as an internal control. Data are presented as the mean ± SD (*n* = 6). Asterisks indicate significant differences between treatments at ***P* < 0.01 (Student’s *t* test).

**Fig. S7** Proposed roles of EcDQD/SDH enzymes in gallate, shikimate, and quinate biosynthesis.
